# Escaping the ordinary: a review of escape rooms in medical and veterinary education

**DOI:** 10.1186/s12909-024-06512-w

**Published:** 2024-12-20

**Authors:** Avis Anya Nowbuth, Vikram Singh Parmar

**Affiliations:** 1 Department of Neuromedicine and Movement Science, Norwegian University of Science and Technology (NTNU), Trondheim, Norway; 2 Pan-African Organization for Health Education and Research (POHER), Missouri, USA; 3 Department of Design, Norwegian University of Science and Technology (NTNU), Trondheim, Norway

**Keywords:** Escape rooms, Medical education, Veterinary education, Active learning, Educational innovation, Systematic review

## Abstract

**Background:**

Escape rooms (ERs), immersive role-playing games that require participants to solve a series of puzzles within a set time to achieve a specific goal, have gained popularity as innovative educational tools.

**Methods:**

A systematic review was conducted using the PRISMA guidelines, a comprehensive search of PubMed, Cochrane, Web of Science, and Scopus, for articles published between inception of journals to April 2024, focusing on the integration, outcomes, and participants’ perceptions of ERs in medical and veterinary education.

**Results:**

A total of 619 articles were retrieved, of which 12 articles met the inclusion criteria for final analysis. These studies focused on medical students and included medical education topics such as nephrology, human physiology, and dermatology. Notably, no ERs focused on the veterinary sector or directly addressed the One Health approach. ERs demonstrated a significant impact on students’ self-reported knowledge, motivation, and collaboration skills gains. Participants reported improved confidence in clinical situations and a greater appreciation for interdisciplinary team dynamics. Most studies yield moderate MERSQI scores and impacts at Kirkpatrick Levels 1 and 2.

**Conclusion:**

ERs increased immediate educational engagement and showed potential in improving an understanding of complex, interrelated health issues. This gap suggests a need for curricula that incorporates ERs to bridge human, animal, and environmental health sectors. The integration of ERs could be instrumental in equipping future prescribers with the interdisciplinary knowledge and skills needed to tackle complex health crises.

**Clinical trial number:**

Not applicable.

**Supplementary Information:**

The online version contains supplementary material available at 10.1186/s12909-024-06512-w.

## Introduction

Traditional learning paradigms, which are often characterized by lectures, repetitive tasks, and memorization, are being increasingly supplemented, and at times replaced, by more dynamic and interactive forms of learning [1]. One such innovative approach is gamification, which integrates game design elements into the learning environment to motivate and engage students [2]. Escape rooms (ERs), a gamification strategy, have been proposed as a novel educational tool [3, 4]. ERs are immersive, scenario-based experiences where participants solve puzzles to accomplish tasks within a set time limit and can be used in education to engage students in active learning, critical thinking, teamwork, and problem-solving [5–7]. ERs have been implemented in a variety of fields; including pharmacy [6, 8], nursing [9], dentistry [10], in different levels of education [11, 12] and for team building, interviews, and skill acquisition in healthcare simulations [13–15]. Intriguingly, there are also a variety of topics that have been addressed using ER, including excessive salt in cardiovascular disease [16], urosepsis [8], mathematics [11], and clinical case scenarios [17]. The diversity of applications of ERs is growing, and the potential for educational innovation approaches is increasing [5], however empirical evidence highlighting the effectiveness of ER in education is still necessary in order for developers, educational curriculum implementers and lecturers to consider the use of game-based learning for students in practice [9, 18, 19].

Game-based learning has shown to be highly effective compared to traditional learning methods [19, 20]. Millennials show a preference for interactive and engaging learning experiences, making ERs a suitable educational tool for this generation [21, 22]. Students are more engaged and exhibit improved learning outcomes with technology and games in various disciplines [2, 20].

At first glance, medical and veterinary students may not have overlapping educational curricula, however within the field of infectious disease, zoonoses, vector-borne diseases and water-borne diseases; antimicrobial resistance (AMR) is prevalent across the human healthcare, animal, and environmental sectors [23]. The One Health approach is “*an integrated*,* unifying approach that aims to sustainably balance and optimize the health of people*,* animals and ecosystems*” [24] and recognizes that the “*health of humans*,* domestic and wild animals*,* plants*,* and the wider environment (including ecosystems) are closely linked and inter-dependent”* [23–25]. As such, ERs are relevant for One Health and AMR particularly because they simulate real-world, collaborative, interdisciplinary problem-solving scenarios that encourage critical thinking and collaboration needed to tackle complex global health issues, like AMR. The WHO has highlighted the collaboration between the sectors when tackling AMR, and multidisciplinary collaboration is necessary in relation to educational initiatives [26–29]. In the context of One Health, specifically medical and veterinary education, ERs could be designed to simulate clinical scenarios, diagnostic challenges, or ethical decision-making situations. This type of active learning could enhance the retention of information, develop practical skills, and improve communication and collaboration among students [20, 30].

**Fig. 1:**
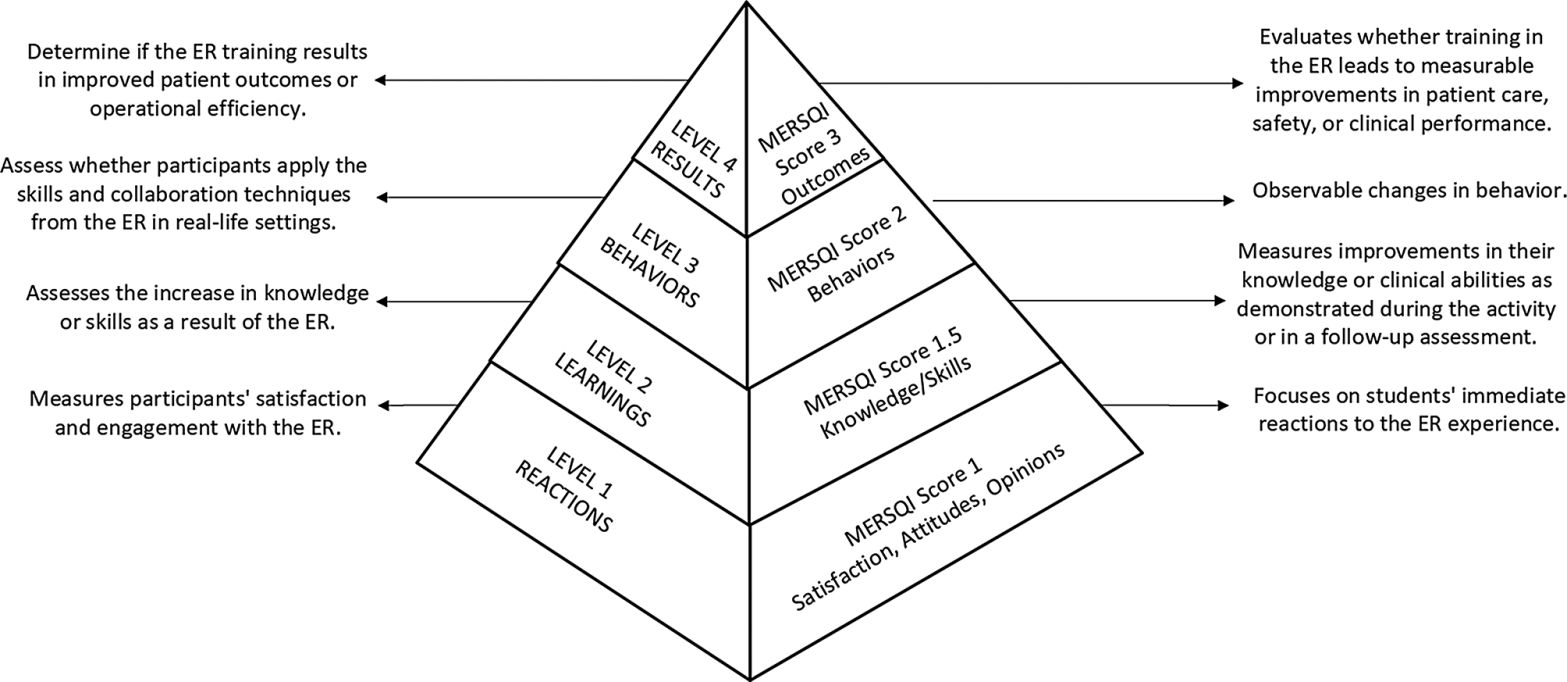
Kirkpatrick’s levels of learning evaluation in relation to the MERSQI outcomes domain. Scoring in the MERSQI represents the points for each outcome’s domain, with higher scores implying more robust outcomes

Although multiple studies have assessed the effectiveness of ERs, no benchmark tool has yet been established for ER educational initiatives. There are, however, a few validated tools that assess educational interventions [5, 7]. A systematic review on ERs for pharmacy students [6], and nurses [9] has been done, and a protocol for ER for medical students has been published [31] but to date, a completed study has not been found. Although there is a review on healthcare professionals and students using ER [7, 32], there is still a need to describe and evaluate ER for medical and veterinary students to guide researchers, game developers and digital designers with the ideal practices for future implementation and evaluation given the students time-constraints in medical and veterinary schools. This review aims to collate the evidence on existing ERs aimed for medical and veterinary students, and assess their effectiveness, approach, and design. The Population, Intervention, Comparator and Outcome (PICO) approach was used to design the research question (RQ) [33]; where population was identified to medical students, veterinary students; intervention: educational ERs; comparator: traditional learning or other educational interventions and the outcome: learning outcomes of educational ERs (knowledge, attitudes, skills developed from the intervention). The specific RQ is *“Are educational escape rooms more effective than traditional learning in improving general learning outcomes among medical or veterinary students?”* The findings of this review will support researchers, educators, game developers, and digital designers create and implement ER within the One Health sectors (Fig. 1).

## Methods

### Study design

**Fig. 2:**
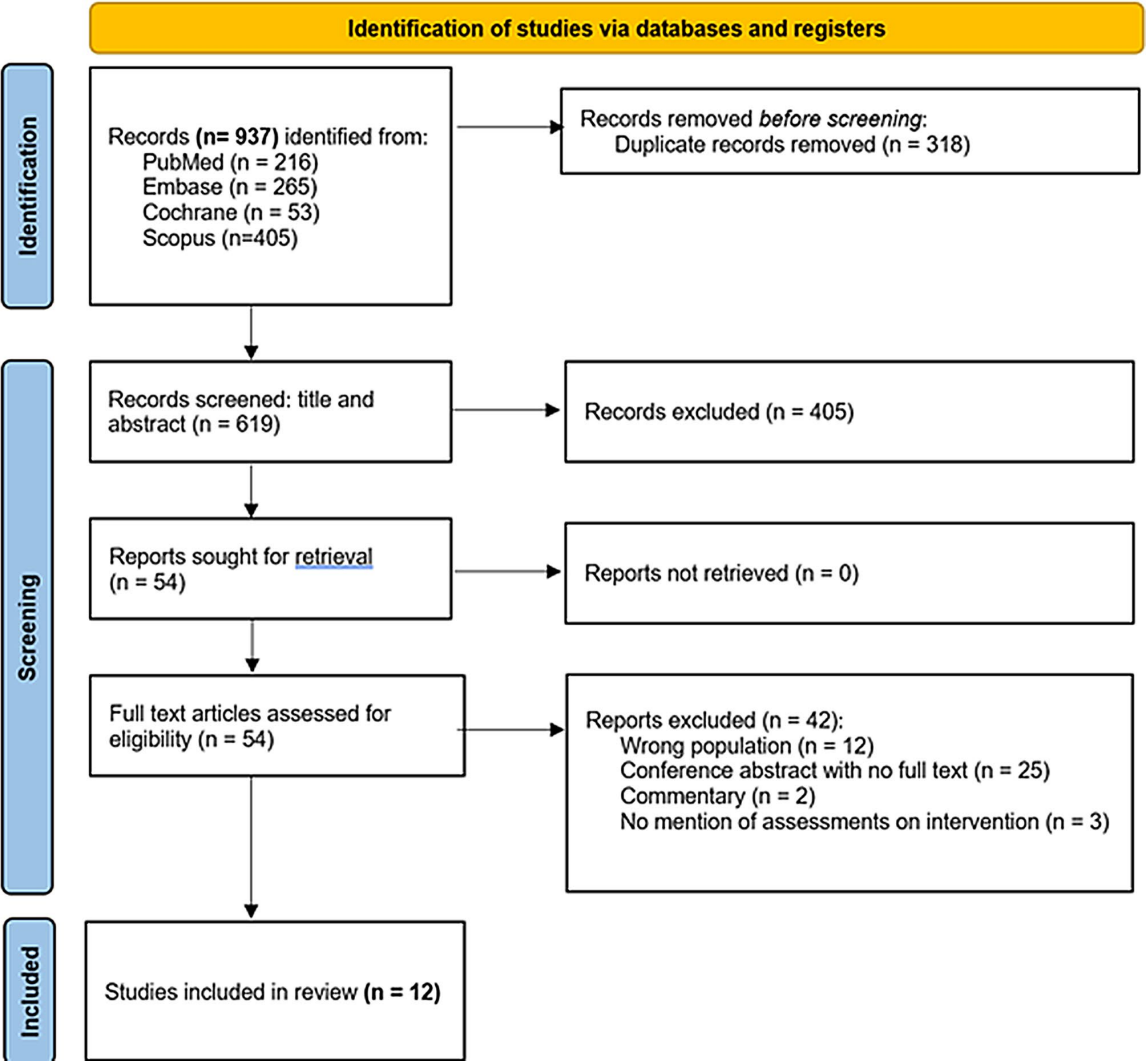
PRISMA flow chart illustrating the study selection process on ERs in medical and veterinary education

A systematic review was performed using the Preferred Reporting Items for Systematic Reviews and Meta-Analysis (PRISMA) (Fig. 2) [34, 35]. An initial scoping search to establish our review aim revealed that there are existing primary studies reporting on ER for training in the pharmacy and nursing professions [6, 7]. Therefore, a study focused on ER use for medical and veterinary students is warranted to assist in the design and influence of future training interventions. Using the guidelines from Randles and Finnegan (2023), systematic reviews allow for the culmination of available evidence to provide insights [36].

### Search strategy

A search strategy was developed using PubMed and Embase (Appendix 1). The search terms [(“*escape room*” OR “*escape game*” OR “escape room*”) AND (“*education*” OR “*teaching*” OR “*training*” OR “pedagog*” OR “*learning outcome*” OR “*Education*, *Professional* [MeSH Terms]” OR “*Teaching* [MeSH Terms]” OR “*Academic Success* [MeSH Terms]” OR “*Academic Performance* [MeSH Terms]”)] were used to search PubMed, Embase, Cochrane and Scopus. Various spellings of the search terms were considered. A Boolean search strategy was done, where the concept terms were combined with ‘AND’ and terms within a concept were combined with the word ‘OR’. The MeSH terms were specified for each database, while the free-test searches remained the same for all databases. No MeSH term existed for ‘escape room’ due to its novelty; however, ‘gamification,’ a related umbrella term, was added to the MeSH library in 2022. The decision to exclude the MeSH term was based on the novelty of the topic, and the lack of articles that were provided when a preliminary search was conducted. Literature search began on 6th March 2024, and completed on 18th April 2024. A total of 937 articles were identified from the four databases. No limitations on publication dates, language or type of paper were set.

### Inclusion and exclusion criteria

Full-text articles addressing the use of an ER for education, specifically for medical and veterinary students were used for the review. No limitations on publication dates were set, and full-text articles had to be published in peer-reviewed journals or international conferences/ workshop proceedings. Table 1 highlights the inclusion and exclusion criteria of the papers that underwent final analysis.

**Table 1:** Inclusion and exclusion criteria

Inclusion criteria	Exclusion criteria
Described the use of ERs as an educational strategy Involved students from medical or veterinary backgrounds Reported on educational outcomes, learner engagement, or both	Not in English Conference abstracts without full texts Only mentioned ERs, but did not provide empirical evidence or evaluation of the intervention Book, book chapters or chapters Studies outside the medical and veterinary fields

### Study selection

AAN, the first author, performed the search, and uploaded the articles to Rayyan, an online tool to facilitate review by helping to organise, screen, and analyse research articles by using features such as collaboration, tagging, filtering and classifying studies according to the inclusion and exclusion criteria thus streamlining the review process [37]. Rayyan was used to remove duplicates and perform independent, blind screening to determine eligibility. Full-text screening of all screened articles to determine legibility for inclusion was done by AAN.

### Data extraction

The data extraction was independently done by AAN on a pre-defined data extraction Excel sheet. Articles that met the inclusion criteria and reported the empirical outcomes of the ER were included in this systematic review. Information extracted included (a) article information (authors, year of publication, location, and specific sites), and (b) study design (cross-sectional design, prospective, randomized controlled trial), and (c) student population (medical or veterinary students), and (d) academic year assessed, year of survey, sample size, type of data, and e) specific data from the studies. Only published data were used in the data extraction and subsequent analysis.

### Quality assessment

The studies included in the analysis were evaluated based on the Medical Education Research Study Quality Instrument (MERSQI) [38, 39]. MERSQI is a 10-intrument evaluation framework, subdivided into six domains: study design, sampling, data type, validity of evaluation instrument, data analysis, and outcome measures; that is used to assess the quality of the medical education research studies [38]. Each domain contributes to the overall score totaling 18, where 3 is the maximum score per domain.

Additionally, we evaluated the studies using Kirkpatrick Model (Fig. 2), framework used to assess the effectiveness of training programs. This model consists of four levels: reaction, learning, behavior, and impact/results [40, 41]. Reaction (level 1) refers to the participants reaction to the intervention (whether the intervention is considered useful, engaging etc.); learning (level 2) refers to the effects of the intervention (whether the intervention imparts knowledge, skills, attitude and confidence on the addressed topic); behavior (level 3) refers to the behavioral changes of the participants (whether the learners apply what they have learned in training); and impact (level 4) refers to the degree to which the intervention has an effect (whether or not the intervention has affected the global public health) [40].

### Data analysis

A narrative synthesis of the articles was conducted using the guidelines from Popay et al. (2006) [42]. A narrative synthesis is an approach to summarize and compare the main features of studies that are included in a review, specifically in the effect of interventions and the factors that shape the implementation of interventions [42, 43]. When applying Popay et al.’s guidelines for synthesis, we defined the understanding of ER and how they promote active learning, engagement, and teamwork. Following this, we conducted a preliminary analysis where we gathered insights from the studies that reported on participants experiences, motivation and perception of benefits when using ER; these were then grouped into themes (engagement, confidence levels, subject/area taught). Additionally, we gathered insights in on the measurable outcomes such as improvements in knowledge, performance on tests, engagement levels (i.e. pre- post-test responses). The quantitative findings are reported in Tables 2 and 3 summarize the both the quantitative and qualitative data. Descriptive statistics, including mean, standard deviation, and ranges, were used to summarize the quantitative data across studies. We calculated the mean improvement in educational outcomes (e.g., knowledge retention, test scores) and used SD to assess the variability of these outcomes across different ER interventions. The range helped provide further context regarding the spread of the results. These statistics allowed us to identify overall trends in the effectiveness of ERs for education. From there, the next step involved examining the relationships between the studies. We looked at how the qualitative themes (e.g., enhanced engagement or improved teamwork) aligned with the quantitative findings. We also assessed the methodological quality of the studies, reported in Table 3, using the MERSQI score, where randomized controlled trials carried a greater score compared to a single site post-interventions study. Numerical summaries (sample size, mean, median, inter-quartile range, types of the evaluation conducted, results on knowledge, effectiveness, confidence levels, and preference of types of education (lectures vs. ERs)) of the study’s characteristics were reported in Table 3 if it was provided in the study, additional qualitative and quantitative data of the intervention’s effects were organized and reported in Table 2 and were further reported according to the Kirkpatrick Model. Overall and domain specific MERSQI scores for the published articles were compared using descriptive statistics. The heterogeneity of outcomes and measurement scales across studies, as well as the limited availability of directly comparable pre- and post-intervention performance metrics, meant that a complete meta-analysis was not able to be done. A combination of narrative synthesis for qualitative outcomes and a descriptive data analysis for quantitative outcomes was conducted (Table 4).

**Table 2:** Characteristics of articles included in this review

No.	Authors	Subject/area	Participants	Methodology	Key findings	Limitations	MERSQI	Kirkpatrick level
#1	Hu et al. (2023)​	Nephrology	52 medical students	Nephrology-themed ER focusing on physiology, pharmacology, pathology, and clinical practice guidelines.	Significant self-reported knowledge improvement; high enjoyability and perceived effectiveness.	Self-reported measures and lack of long-term knowledge retention assessment.	9	Level 1: Reaction Level 2: Learning
#2	Kinio et al. (2019)​	Vascular surgery	13 medical students	Vascular Surgery-themed ER to engage students in both knowledge-based problems and technical skills.	Increased motivation and consolidation of knowledge; positive reception towards practical exercises.	Lack of control group and evaluation focused on group knowledge rather than individual knowledge.	7,5	Level 1: Reaction Level 2: Learning
#3	Backhouse & Malik (2019)	Patient safety	19 medical students	Gamified simulation with an ER designed around clinical tasks and communication skills to enhance patient safety understanding.	High student engagement and confidence in applying patient safety concepts.	Small sample size and limited evaluation to immediate feedback.	8,5	Level 1: Reaction
#4	Martin & Gibbs (2022)	Preclinical preparation	107 medical students	A 90-minute ER orientation activity for preclinical medical students was designed where a routine clinic visit scenario, where students had to perform tasks related to basic vital sign measurement, physical exam maneuvers, and patient assessment using a patient manikin.	Majority found the ER effective and increased their confidence in using basic equipment and assessing vital signs. Teamwork and communication among students were highlighted and impacted their performance. Facilitators were vital in the process of guiding students through the ER.	Low response rate to the second questionnaire. Barriers such as class size, facilities, and time constraints were noted. Some students did not get to practice all tasks due to the team-based nature of the activity	9,5	Level 1: Reaction Level 2: Learning
#5	Faysal et al. (2022)	Dermatology	97 medical students	Compared medical ER with case-based learning (CBL) for teaching clinical dermatology.	ER as effective as CBL, with higher student performance improvement in ER group.	No long-term knowledge retention analysis; effectiveness based on immediate post-activity assessment.	12,5	Level 2: Learning
#6	Guckian et al. (2019)	Dermatology	16 medical students	Dermatology-themed ER to enhance understanding and interest in dermatology.	Improved perceptions of dermatology, increased interest in specialty, and enhanced confidence in identifying skin conditions.	Small sample size, non-compulsory participation may introduce selection bias.	8	Level 2: Learning
#7	Carrasco-Gomez. et al. (2023)	Human physiology	245 medical students	Peer-to-peer designed cardiorespiratory themed ER for Human Physiology.	Significant increase in final exam marks for participants vs. non-participants; positive qualitative feedback.	Analysis limited to immediate academic performance; lacks long-term retention assessment.	9,5	Level 1: Reaction Level 2: Learning
#8	Moore & Campbell (2021)	Interprofessional practice	9 medical students	ER followed by an educational workshop focusing on teamwork and interprofessional practice.	Improved knowledge on interprofessional practice and teamwork; high engagement and positive feedback on the learning method.	Sample not reflective of the health workforce profile; immediate post-workshop knowledge assessment does not confirm long-term learning.	9	Level 2: Learning
#9	Akatsu et al. (2022)	Medical interview and physical examination	140 medical students	Course including ERs for final assessment of medical interview and physical examination skills in Japan.	High ratings for medical interview skills and successful completion of physical examination tasks in ERs; enhanced student motivation and interest.	Descriptive study with both quantitative and qualitative data but no control group for comparison.	8	Level 1: Reaction
#10	Cantwell et al. (2022)	Emergency medicine	134 medical students	Virtual escape box in emergency medicine clerkship to teach chest pain and abdominal pain, compared to traditional flipped classroom format.	High engagement, satisfaction, and preference for the escape box over traditional methods; better ratings for didactics compared to flipped classroom.	Lack of pre- and post-game testing to assess knowledge acquisition.	8,5	Level 1: Reaction
#11	Liu et al. (2020)	Paediatric radiology	19 medical students	A paediatric radiology-themed ER designed around key learning objectives including radiation protection, fracture detection, and emergency findings on paediatric chest radiographs.	Significant improvement in paediatric radiological knowledge post-session; high levels of enjoyment and preference for ER-based learning.	Small sample size; mixed levels of prior radiological knowledge among participants.	8,5	Level 2: Learning
#12	Moffett et al. (2023)	Uncertainty management	50 health professional students − 22 medical students	Digital Educational ER (DEER) to facilitate learning around uncertainty management in clinical settings.	Facilitated learning around managing uncertainty, with an emphasis on collaborative problem-solving and reflection.	Focus on the qualitative data collection with a small sample size; specific to the transition from pre-clinical to clinical training.	8	Level 2: Learning Level 3: Behavior

**Table 3:** Study interventions data

No.	Authors	Subject/area	Samsam size (*n*)	Type of study	Evaluation design	Pre-test scores	Post-test scores
1	Hu et al. (2023)​	Nephrology	52	Mixed methods	Wilcoxon signed-rank test for significant difference between pre-and post-participation responses. Spearman correlation to compare working alone or with others’ impact.	Before the ER activity, students rated their understanding of renal physiology, pharmacology, pathology, and clinical practice guidelines on a five-point Likert scale, with lower scores indicating less knowledge	80.8% rated the ER as more effective than traditional lectures; 73.1% rated the ER as more effective than textbooks; 69.2% rated the ER equally effective to third-party board prep resources; 51.9% rated the ER equally effective to the school PBL tutorials. 95% rated the ER very effective in integrating information from all first-year courses so far. 84.6% rated the ER very effective in helping identify rooms for improvement.
2	Kinio et al. (2019)​	Vascular surgery	13	Qualitative	Post-experience survey results on knowledge retention, format appropriateness, and role encouragement.	N/A	75% of respondents felt the experience improved their ability to retain information, and 92% found the format appropriate for testing knowledge, indicating a positive learning outcome. 92% gained interest in vascular surgery following the ER. 100% of participants found the ER enjoyable.
3	Backhouse & Malik (2019)	Patient safety	19	Qualitative	Feedback form with outcome measures and student evaluations.	N/A	100% agreeing or strongly agreeing that they gained new knowledge and skills, and 100% feeling confident or very confident in applying what they learned in the future
4	Martin & Gibbs (2022)	Preclinical preparation	107	Qualitative	DASH33 and SET-M34 surveys were used to evaluate effectiveness.	Before participating in the ER activity, students were surveyed to assess their confidence levels in performing critical actions related to patient assessment and equipment use.	82% rated the ER as highly effective or very effective for preparation and, 80% highly effective or very effective in acclimating them to the simulated patient
5	Faysal et al. (2022)	Dermatology	97	Quantitative	Wilcoxon Signed Rank test for association between teaching strategies’ effectiveness Shapiro Wilk test for data distribution analysis	Medical ER Group (Group A): Median of 58 with IQR of 16 Case-Based Learning Group (Group B): Median of 56 with IQR of 20.5	Medical ER Group (Group A): Median of 86 with IQR of 19.2 Case-Based Learning Group (Group B): Median of 83 with IQR of 13.5
6	Guckian et al. (2019)	Dermatology	16	Qualitative		43.8% preferred lectures; 31.3% preferred self-directed learning; 25% preferred small group teaching. 31.3% felt confident prior to the ER. 12.5% confidence in approaching a systematic skin examination prior to ER	100% enjoyed the ER; 81.3% felt confident following the ER. 68.8% confidence in approaching a systematic skin examination following the ER. 94% reported that they gained interest in dermatology following the ER
7	Carrasco-Gomez. et al. (2023)	Human physiology	245	Qualitative	Descriptive analysis with mean, standard deviation, minimum, and maximum. Parametric unpaired t-test and non-parametric Mann-Whitney U test. Bonferroni correction for seven independent comparisons.		59% felt prepared to solve the cardiovascular stage, 26% felt prepared to solve the respiratory stage, and 21% felt confident to solve the final stage. 98% of students found the activity positive and advantageous for their learning. Students who completed the ER activity had better overall exam and final marks compared to the participants who did not participate in the ER.
8	Moore & Campbell (2021)	Interprofessional practice	9	Qualitative	Descriptive analysis for quantitative data, paired-samples t-tests for comparisons.		Results from overall sample (50), participant satisfaction was rated as 9.06/10; 90% ranked the ER their preferred format for learning about teamwork and interprofessional practice. 50% of participants mentioned the novel nature of learning in the ER.
9	Akatsu et al. (2022)	Medical Interview and Physical Examination	134	Mixed methods		Not provided	Not provided
10	Cantwell et al. (2022)	Emergency medicine	140	Quantitative	Likert scale surveys to assess engagement, learning, satisfaction, and application.	49 (confidence out of 100)	73 (confidence out of 100)
11	Liu et al. (2020)	Pediatric Radiology	19	Qualitative	Descriptive analyses with total, mean, and range of feedback scores. 8-question single best answer (SBA) test for knowledge improvement.	46.25% on knowledge scores (single best answer test) with a range of 2–6 correct answers	91.25% on knowledge scores (single best answer test) with a range of 4–8 correct answers and, two weeks after the teaching session, the average score remained at 91.25%, with a range of 5–8 correct answers, indicating sustained knowledge retention
12	Moffett et al. (2023)	Uncertainty management	50	Mixed methods	One-sample t-test, descriptive statistics, Cronbach’s coefficient alpha, Shapiro-Wilks test Reflexive thematic analysis approach, NVivo 12 software pre-and post-intervention surveys, paired-design t-test		

**Table 4:** Critical appraisal: MERSQI scores

Domain	Item		Item Score	Hu et al. (2023)​	Kinio et al. (2019)​	Backhouse & Malik (2019)	Martin & Gibbs (2022)	Faysal et al. (2022)	Guckian et al. (2019)	Carrasco-Gomez. et al. (2023)	Moore & Campbell (2021)	Akatsu et al. (2022)	Cantwell et al. (2022)	Liu et al. (2020)	Moffett et al. (2023)
Study Design		Single group cross-sectional or single group post-test only	**1**		1	1	1								
		Single group pre and post-test	**1**,**5**	1,5					1,5		1,5	1,5	1,5	1,5	1,5
		Non-randomized, 2 group	**2**							2					
		Randomized controlled experiment	**3**					3							
Sampling	Institutions	Single institution	**0.5**	0,5	0,5	0,5	0,5	0,5	0,5	0,5	0,5	0,5	0,5		0,5
		Two institutions	**1**											1	
		More than 2 institutions	**1**,**5**												
	Response Rate	Not applicable	**n/a**												
		Response rate < 50% or not reported	**0.5**	0,5	0,5				0,5	0,5	0,5	0,5			0,5
		Response rate 50–74%	**1**											1	
		Response rate ≥ 75%	**1**,**5**			1,5	1,5	1,5					1,5		
Type of Data		Assessment by study participant	**1**	1	1	1	1		1	1	1	1	1	1	1
		Objective measurement	**3**					3							
Validity of Evaluation Instruments’ Scores	Internal Structure	Not reported	**0**	0	0	0	0	0	0	0	0	0	0	0	0
		Reported	**1**												
	Content	Not reported	**0**	0	0	0			0		0	0	0		
		Reported	**1**				1	1		1				1	1
	Relationships to other variables	Not reported	**0**		0	0	0	0	0	0	0	0	0	0	0
		Reported	**1**	1											
Data Analysis	Appropriateness of analysis	Data analysis inappropriate for study design or type of data	**0**												
		Data analysis appropriate for study design and type of data	**1**	1	1	1	1	1	1	1	1	1	1	1	1
	Sophistication of analysis	Descriptive analysis only	**1**												
		Beyond descriptive analysis	**2**	2	2	2	2	2	2	2	2	2	2	2	2
Outcome		Satisfaction, attitudes, perceptions, opinions, general facts	**1**										1		
		Knowledge, skills	**1**,**5**	1,5	1,5	1,5	1,5	1,5	1,5	1,5	1,5	1,5		1,5	1,5
		Behaviors	**2**												
		Patient/health care outcome	**3**												
Total				**9**	**7**,**5**	**8**,**5**	**9**,**5**	**13**,**5**	**8**	**9**,**5**	**8**	**8**	**8**,**5**	**10**	**9**

## Results

The final search identified 937 articles (Fig. 2) from inception of the database to March 2023. Three-hundred-and-eighteen articles were identified as duplicates and removed using Rayyan. Six-hundred and nineteen articles underwent title and abstract screening, of which 54 articles remained for full-text review. Table 2 summarises the characteristics of the analysed articles, and a full list of included articles is provided in Tables 2 and 3.

### Study characteristics

A total of 12 articles evaluated the educational aspect of ERs focused for medical and veterinary students and is summarised in Table 1. Seven articles were published in journals dedicated to medical education, while the remaining five appeared in specialized journals relevant to their specific fields. Additionally, the publication trends highlight the novelty of ERs, as most of the studies were conducted and published between 2019 and 2023. All included studies took place in the human healthcare sector only. No studies were found for the veterinary/ animal healthcare sector. Within the healthcare space, topic areas that were addressed were nephrology [44], vascular surgery [45], tasks for clinical practice (patient safety, preclinical preparation, interprofessional practice and medical interviews with physical examinations) [46–49], human physiology [50], emergency medicine [51], paediatric radiology [52] and uncertainty management [53]. There were two dermatology themed ER [21, 54].

**Fig. 3:**
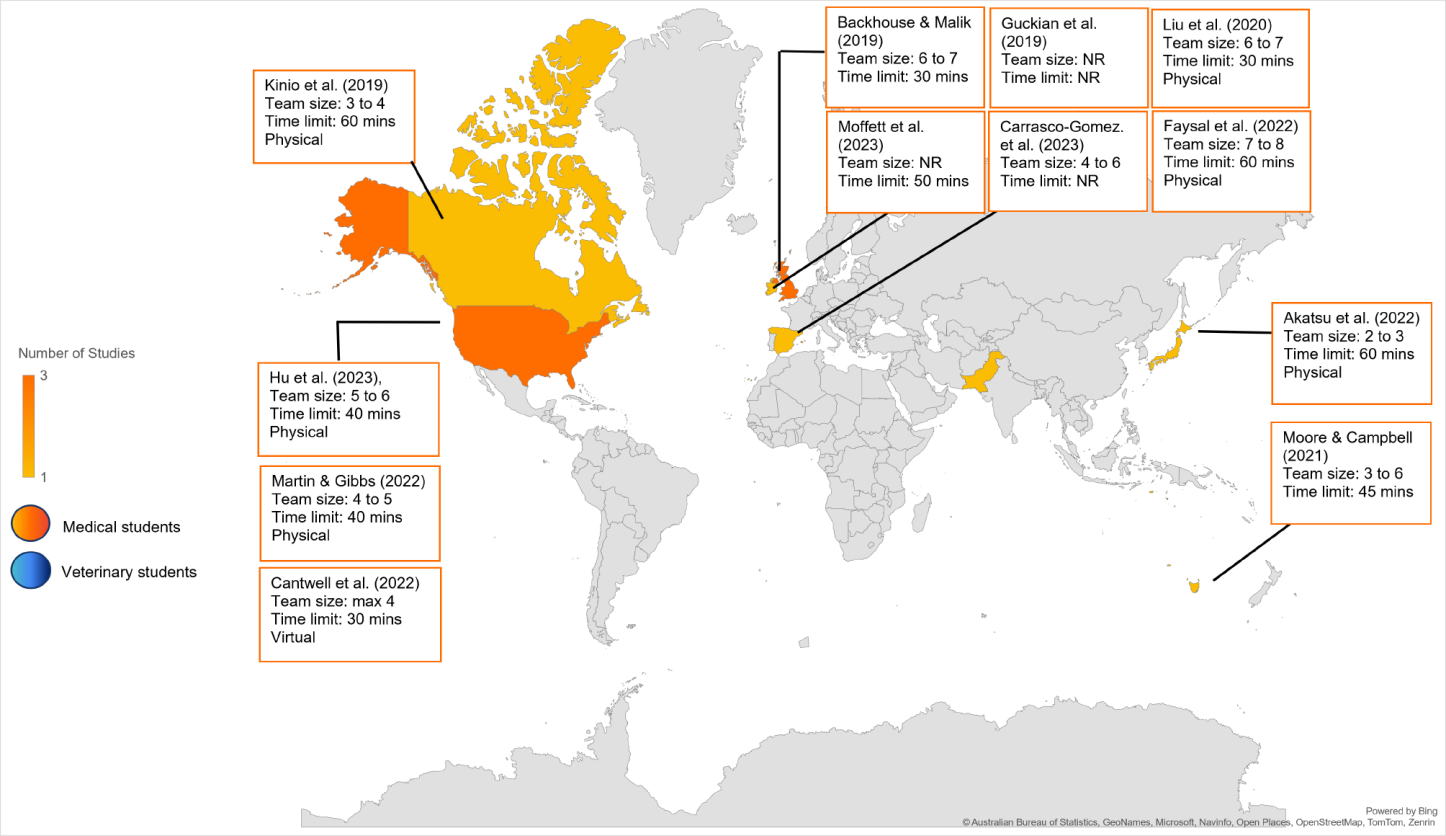
Characteristics of included articles

The countries that reported quantitative and qualitative relevant data are shown in Fig. 3. Most (*n* = 11) ER experiments were held in university settings in high-income countries (HICs) and took place in a European setting (*n* = 5) followed by North America (*n* = 4), two in Asia, and one in Australia. Africa and South America did not record any studies. All but two ERs took place in-person, with the online versions taking place in Zoom breakout rooms [51, 53].

Majority of the ERs were team-based (*n* = 8). Teamwork was usually done in groups of less than three (*n* = 1), up to four (*n* = 1) up to five (*n* = 1) up to six (*n* = 3) and up to seven (*n* = 2). Faysal et al., (2022) had the largest team, consisting of seven to eight members per team [54]. The remaining studies had not reported team sizes or had participants attempt the ER individually. Moore and Campbell et al. (2021) and Moffet et al., (2023) had teams with participants from different disciplines.

### In-game mechanics

To escape the ER, participants had to solve riddles, puzzles, and coded messages to achieve the learning objective set by the game developers. One of the ERs had sequential puzzles, where participants had to complete one puzzle to gain a clue to the next puzzle in the sequence [44]. Some (*n* = 8) had open pathways where participants completed individual tasks, providing clues needed to complete a final puzzle to successfully escape the room. Three ER were tasks based on clinical and non-clinical cases, normally conducted in a simulation room [46, 47, 49].

### MERSQI evaluation

The maximum score an article could have received when undergoing MERSQI quality assessment was 18. MERSQI scores ranged from 8 to 13,5 (Table 3). The mean MERSQI score was 8.92, and a standard deviation of 1.61. The domain *data analysis* consistently scored 3/3 across all studies, while other domains such as *study design* (1.54/3) and *sampling* (1.42/3) showed more variability. Single group pre and post-test study design were the most prevalent (*n* = 7), single group post-test only (*n* = 3), and non-randomized, 2 groups (*n* = 1). Only one study was scored as a randomized controlled trial [54]. All but one study took place in a single institution, whereas Liu et al. deployed their ER in two institutions [52].

### Kirkpatrick evaluation

All the studies had a level of evaluation post-intervention. Six studies had qualitative study designs, with confidence levels, and feedback about the ER was the primary focus of the study. Two articles had quantitative data and performed statistical tests to determine the effect of the intervention. Four articles employed both a quantitative and qualitative design, providing information about the ER, its’ effects and whether they improved confidence, perceptions, or attitudes about the presented topic. The educational outcomes have been reported using the Kirkpatrick Model [40].

### Learning outcomes

Table 1 summarizes the outcomes, and key findings from specific studies are highlighted below.

Hu et al., (2023) [44] implemented a nephrology-themed ER as an innovative educational modality for pre-clinical medical students. The ER was well-received by participants, with high ratings for effectiveness in integrating information and identifying areas for improvement in nephrology topics. Most students found the ER very enjoyable and rated it as more effective than traditional lectures and textbooks. Inter- and intraclass collaboration was also noted, with most students working in groups rather than alone. The study highlighted significant improvements in student understanding of renal physiology, pharmacology, pathology, and clinical guidelines after participating in the ER. Overall, the findings suggest that ERs can enhance peer-to-peer interactions, improve knowledge acquisition, and provide a unique and engaging learning experience in medical education. Hu et al., (2002) recommends additional research to explore the full educational impact of ERs on preclinical topics. The overall MERSQI score for this paper summed to 9/18. The study assessed the Kirkpatrick Level 2: Learning. This is indicated by the significant improvement in self-reported knowledge in renal physiology, pharmacology, pathology, and clinical practice guidelines after participating in the ER. Participants’ reactions, which correspond to Kirkpatrick Level 1: Reaction, were also measured, as most students found the ER to be more effective than traditional lectures and textbooks, and more enjoyable.

Kinio et al., (2019) [45] implemented an ER activity in vascular surgery education for medical students, incorporating knowledge-based problems, and technical skills. Thirteen medical students participated in the activity, divided into four groups. Results showed that teams using collaborative strategies completed the activity successfully with an average time of 53.6 min. Participants reported increased motivation to prepare beforehand, consolidation of knowledge, and enjoyment of practical exercises. The experience also encouraged the use of Canadian Medical Education Directives for Specialists (CanMEDS) roles. Limitations included the small sample size and the labor-intensive nature of the activity. Overall, the study demonstrated the educational potential of ERs in engaging medical students and enhancing their learning experience in vascular surgery. The study scored 7.5/18. The study measured how much students enjoyed the ER, and whether they felt motivated to learn. This links to the Level 1: Reaction of the Kirkpatrick model. Furthermore, the students were assessed post-intervention, where an increased consolidation of knowledge was recognized, corresponding to the Kirkpatrick Level 2: Learning.

Backhouse and Malik (2019) [46] discusses the use of ER simulations as a novel approach to teaching patient safety and human factors to medical students. The simulation involved students working together to solve clinical challenges within a limited time frame, promoting teamwork, communication skills, and active participation. The session received positive feedback from students, who found it engaging and valuable for learning. The ER experience is followed by a structured debriefing session to reflect on the learning points and experiences. The article highlights the potential of ERs as experiential learning tools in medical education and suggests further evaluation to compare their effectiveness with traditional teaching methods. The total MERSQI score summed to 8.5/18. In this study, only the learner satisfaction and reaction were assessed, corresponding to Level 1: Reaction on the Kirkpatrick model.

Martin and Gibbs (2022) [47] used an ER activity to familiarize preclinical medical students with the simulated medical environment. The activity aimed to enhance students’ comfort levels with basic equipment, manikin features, and patient assessment skills. Facilitators included faculty who had prior experience in simulation teaching. The ER sequence included tasks related to equipment use and manikin features, with faculty assessing students’ performance based on a critical actions’ checklist. Debriefing sessions focused on reinforcing learning points and addressing any uncertainties. The activity was found effective in boosting student confidence and readiness for simulated patient encounters, as well as promoting teamwork skills. Limitations included a low response rate to post-activity questionnaires and challenges in ensuring all students completed the tasks. Overall, the ER format proved beneficial in preparing students for simulation-based learning experiences in a medical setting. The overall MERSQI score was 9.5/18. Using the Kirkpatrick model, this paper primarily addresses Level 2: Learning, as it evaluates the increase in students’ knowledge and confidence in using simulation equipment and understanding the simulation manikin after participating in the ER activity. Level 1: Reaction is also assessed, with students reporting their perceptions of the ER’s effectiveness and their engagement with the activity.

Faysal et al., (2022) [54] evaluated the effectiveness of using a medical ER compared to case-based learning (CBL) in teaching clinical dermatology to undergraduate medical students. A total of 97 students participated, with 48 in the medical ER group and 49 in the case-based learning group. Both groups underwent pre-tests and post-tests to assess their learning outcomes. The results showed that the medical ER was equally effective as case-based learning, with a significant increase in post-test scores in the ER group. The study highlighted the potential of the medical ER as an engaging and effective teaching tool, offering students opportunities to enhance their non-technical skills in a stress-free environment. The total MERSQI score was 12.5/18, the highest score in this review. Faysal et al., (2022) focused on Level 2: Learning by measuring the increase in knowledge and skills of medical students through pre-test and post-test assessments. Level 1: Reaction is partially addressed as student engagement and motivation was assessed. The second study that used dermatology as the subject/ area of focus for the ER was Guckian et al. (2019) [21]. Guckian et al. (2019) evaluated the effectiveness of an ER game in enhancing undergraduate medical students’ perceptions of dermatology. Students participated in the ER after attending a lecture on dermatology, and their perceptions and confidence in the subject were measured before and after the game. Results showed that the ER experience was positively received, with students reporting increased interest in dermatology and improved confidence in performing skin examinations. The study suggests that ERs could be a low-resource, learner-centered educational tool to address misconceptions and barriers related to the field of dermatology. The total MERSQI score was 8/18. Students’ feedback and the increase in their desire to experience more dermatology suggest a positive change in attitudes and confidence, corresponding to the Level 2: Learning. Although the study collected qualitative data on student perceptions and confidence, it did not specifically measure behavior change or results.

Carrasco-Gomez. et al. (2023) [50] designed an ER for medical students to improve their learning of human physiology, with a special emphasis on cardiorespiratory systems. The study utilized a quasi-experimental design where 245 s-year medical students participated in the peer-to-peer designed ER after completing their theoretical and practical courses, and their performance was compared to non-participants. Participants of the ER scored significantly higher in the final exam, particularly in cardiovascular and respiratory physiology, compared to non-participants. Despite over 70% feeling unprepared for the tasks, 98% of students found the activity beneficial, and most preferred this peer-to-peer learning method over traditional teaching. The total MERSQI score was 9.5/18. Carrasco-Gomez. et al. (2023) addresses Level 2: Learning since it measures the increase in knowledge and skills of the medical students who participated in the ER; this was evident in their higher scores compared to those who did not participate in the ER. The qualitative feedback from students, that indicate their perceived benefits and how they received the ER can be classed as Level 1: Reaction.

Moore and Campbell (2021) [48] evaluates the effectiveness of an ER as a learning tool for interprofessional practice (IPP) among health professional students. Using a mixed methods approach, 50 students from various health disciplines participated in the ER, of which 9 were medical students. The ER was followed by an interactive session to reinforce IPP concepts; the study used pre-post design to measure changes in knowledge and attitudes. The post-intervention results indicated an increase in IPP knowledge, with students recognizing the importance of teamwork and clear communication in healthcare settings. The main limitation was the sample size and participants are not reflective of wider health workforce; and the testing was limited to the immediate knowledge assessment only. The total MERSQ I score was 8/18. This study measures the increase in knowledge and skills of the students through pre-post session self-rated changes and reflective evaluations, aligning with Level 2: Learning. While there is an element of Level 1: Reaction, through the collection of qualitative data on student engagement and perceptions of the ERs effectiveness, the paper does not explicitly measure satisfaction or participant reactions.

Akatsu et al. (2022) [49] evaluated the impact of teaching medical interviews and physical exams from the start of medical school in Japan, and incorporated the ER as the final assessment. Physicians assessed the interview skills of 140 first-year medical students, while the ER, completed in teams, tasks evaluated physical examination skills. An average of 89% of ER teams completed all tasks correctly within the time limit. Students’ self-confidence in physical examination skills increased from 49 to 73 out of 100 after participating in the ER. The ER reportedly enhanced student motivation and was considered interesting and useful by 99% of respondents. The total MERSQI score was 8/18. The study demonstrated Level 1: Reaction, indicating that students felt their skills and confidence improved after the ER assessment, reflecting on their learning experience positively.

Cantwell et al. (2022) [51] implemented a virtual ER to train emergency medicine to medical students, with an emphasis on chest and abdominal pain. The ER was also compared to a traditional classroom method. A virtual ER game was designed and incorporated into a two-week emergency medicine clerkship, consisting of PDF cases with puzzles, and its effectiveness was evaluated through Likert scale surveys. Out of 134 learners, a significant majority found the ER engaging, informative, satisfying, and applicable to their knowledge, with a desire for more gamified education. The ER received higher scores for clarity and instructional material effectiveness than the flipped classroom format. The primary limitation of this study was lack of pre- and post-game testing to assess knowledge acquisition. The total MERSQI score was 8.5/18. Level 1: Reaction is assessed with surveys measuring participants’ satisfaction, engagement, and perceptions of the educational method.

Liu et al. (2020) [52] developed a paediatric radiology themed ER for 19 undergraduate medical students and assessed its effectiveness in improving knowledge and student satisfaction. Key learning objectives from the established curriculum were integrated into the ER. Students’ knowledge was tested pre- and post-intervention; followed by a two-week follow-up. The ER was well-received, with high satisfaction scores and a preference for this interactive teaching method over traditional lectures; students showed a significant improvement in pediatric radiology knowledge. The main limitation of this study was the small sample size, and mixed levels of prior radiological knowledge among participants. The total MERSQI score was 10/18. This paper demonstrates learning through significant improvement in pediatric radiological knowledge post-session. The emphasis on knowledge gain in a specific area of medical education suggests a focus on Level 2: Learning.

Moffett et al. (2023) [53] investigates the use of a digital educational ER to help medical students manage uncertainty when transitioning from classroom to clinical settings. A mixed methods pilot test was conducted with 22 medical students participating, utilizing both qualitative (focus groups, game-play observations) and quantitative (questionnaires) data collection methods. Most students (82%) felt that the ER supported their learning around uncertainty, providing new insights and strategies for managing it, such as teamwork and embracing different perspectives. The main limitation of this study was a focus on qualitative data and participants specific to pre-clinical to clinical transition. The total MERSQI score was 9/18. Level 2: Learning was he primary focus of this study as it evaluates the increase in knowledge and skills of medical students in managing uncertainty using ER. Evidence of Level 3: Behavior is suggested through the reported changes in students’ approaches to uncertainty, indicating potential behavioral changes post-intervention.

## Discussion

The findings indicate that ERs can increase student engagement, motivation, and knowledge, with several studies highlighting improvements in performance, confidence, and interest in specific medical areas such as nephrology, dermatology, and emergency medicine. Many studies report high levels of student enjoyment and positive feedback for the learning methods. Common limitations include small sample sizes, lack of control groups, and a lack of long-term knowledge retention assessment. The Kirkpatrick levels across these studies generally range from Level 1: Reaction to Level 2: Learning, with one study achieving Level 3: Behavior. The MERSQI scores vary from 7.5 to 13.5, indicating variable study quality.

A novel aspect in this approach is the use of an educational ER format to facilitate active learning in medical education, which represents a shift from traditional didactic teaching methods to more interactive and engaging forms of learning. ER can be considered a novel approach in learning since they employ “active learning” – a process in which the students engage directly with the material through activities that promote critical thinking, problem solving and the application of knowledge [30, 55, 56]; a contrast to traditional lectures where “passive learning” – students receive information from a lecturer without direct engagement – occurs [30, 55, 56]. Some studies incorporated unique themes, such as peer-to-peer designed rooms or digital formats, that emphasize the evolving role of technology and peer learning in medical education. The findings are relatively novel in demonstrating that ERs can effectively teach complex medical concepts and skills, offering an innovative angle on curriculum design and student assessment in the medical education field.

Educational ERs, as novel initiatives, advance SDG4’s objectives by enhancing quality education and inclusivity, particularly through active and engaging learning environments that cater to diverse learning needs [57, 58]. The concentration of studies in HICs and university settings raises questions about the accessibility and applicability of ERs across diverse educational and healthcare contexts. The absence of studies from Africa and South America, and the limited focus on veterinary medicine, suggests a need for broader exploration, collaboration and better interdisciplinary approaches when addressing topics in One Health education [26, 59, 60]. Studying how ER are used in diverse contexts is important to understand *how* ER can be modified and applied successfully within these different contexts – researchers can better tailor tools to accommodate a broader range of students such as those with different needs (disabilities, gender, age) and avoid stereotypical designs [61, 62]. Additionally, since ER incorporate various game-design elements (visual, auditory) not all students engage with these in the same way, this testing in various contexts allow for how different groups learn and interact – thereby providing feedback for researchers to improve ERs [63]. Furthermore, adapting ER to be inclusive and respectful of different cultural contexts is vital; and studying how these are tested in various cultural contexts may highlight where modifications can be made to ensure relevance and thus, engagement [18]. These factors will affect the effectiveness of ER in achieving the learning outcomes and these considerations highlight the need for ongoing research and adaptation.

Additionally, there are various types of ERs that can be employed: These include physical ERs, where students solve puzzles in person using tangible objects [64]; digital or virtual ERs, hosted entirely online for remote learning [65]; and hybrid ERs, which blend physical and digital elements [66]. Additionally, subject-specific ERs can be tailored to focus on specific academic areas [64]. There are also problem-based ERs, which encourage interdisciplinary problem-solving, and collaborative ERs, designed to foster teamwork and communication among students [67].

All the studies were focused on human healthcare sector only, with a variety of topic addressed ranging from general clinical practice (medical interview and physical examinations) to subspecialities (pediatric radiology and vascular surgery). The medical curriculum is considered bulky, with complex topics and in need for continuous adaptation [68]. The main challenge educators have related to what areas need to be prioritized, and novel innovative methods are necessary [69]. Several studies have highlighted the effect of gamification on student motivation, collaboration, engagement and improvement of skills and knowledge in different fields [2, 70–73]. However, the field needs continuous development and empirical evidence to support gamification integration within educational curriculum [74]. The design of games that address broad global health threats, like antimicrobial resistance, can effectively break complex topics into manageable ‘bite-sized’ units. This approach not only increases student motivation and desire to learn but also enhances knowledge acquisition and the development of skills and attitudes. Such targeted games could significantly enrich medical and veterinary curricula [26, 75, 76].

A notable pattern across these studies is the effectiveness of ERs in promoting retention and application of knowledge in certain medical subjects. ERs themed around nephrology, dermatology, and patient safety have shown to be beneficial in helping students integrate complex information and apply it in a clinical context [21, 44, 46]. Faysal et al. (2022) demonstrated that medical students in the ER group performed better than those in traditional case-based learning settings [54], while Liu et al. (2020) showed sustained knowledge retention in pediatric radiology weeks after the intervention, which is promising for long-term learning outcomes [52]. Alexandropoulou and Tsezou (2023), Alabdulaziz (2023) and Rodríguez-Ferrer et al. (2022) corroborate that the immersive and interactive nature of ERs enhances memory retention, motivation and improvement in skills or knowledge, perhaps due to the added emotional engagement and the active recall processes involved [11, 65, 77]. Students’ reported an increase in confidence in performing critical actions related to patient assessment and equipment use [47], in approaching a systematic skin examination [21] and in applying what they learned about patient safety [46]. Confidence levels are crucial for medical students as they reflect knowledge self-monitoring, efficiency and can indicate appropriate behaviors in clinical practice [78, 79]. Enhancing confidence levels through targeted training and support is essential for ensuring competent and effective medical professionals.

High engagement levels, observed in studies such as that by Martin and Gibbs (2022), reinforce the idea that active participation in learning tasks increases student interest and investment in the material [30]. Much like PBL scenarios, ER require active engagement which is a fundamental principle of active learning. Active participation contrasts with the passive absorption of knowledge that characterizes traditional lecture-based learning [55]. This can lead to deeper processing of information and a more meaningful learning experience, as students are not passively receiving information but are discovering and applying knowledge through problem-solving [30, 80]. Most ERs were designed to be team-based, promoting collaboration and interprofessional skills. This aspect is particularly highlighted in studies by Moore and Campbell (2021) and Moffett et al. (2023), where ERs facilitated learning around teamwork and managing uncertainty, essential competencies in healthcare settings. Additionally, incorporating Communities of Practice (CoPs) can further extend the collaborative nature of ER-based learning by creating ongoing professional networks where educators and practitioners can share expertise, solve problems, and engage in sustained professional development [81, 82]. By fostering an immersive and interactive environment, ERs can complement traditional medical education by providing a more immersive, engaging, and memorable learning experience that may lead to better retention and application of knowledge, as well as the development of teamwork and communication skill [30, 80, 83]. ERs are perceived by students as an enjoyable and effective way to learn complex medical material and develop clinical skills [22], potentially leading to improved educational outcomes compared to traditional teaching methods. From a pedagogical standpoint, these findings support the incorporation of active learning strategies into medical education.

Limitations across these studies are noticeable, with several common themes. A lack of long-term knowledge retention assessment is a recurring issue. This raises the question of whether the immediate benefits in terms of engagement and short-term knowledge gains translate to long-lasting educational outcomes. The variability in MERSQI scores, particularly in study design and sampling, highlights the methodological limitations within the current body of literature. The predominance of single-group designs and the lack of randomized controlled trials limit the generalizability of findings. Additionally, small sample sizes and the absence of control groups in several studies limit the generalizability of the results. The lack of rigorous assessment methods further clouds the efficacy claims, as it’s unclear how these activities compare to traditional educational methods in objective terms. Despite these limitations, the consistently high scores in the MERSQI and Kirkpatrick Level assessments indicate that ERs are well-regarded as educational tools. Most studies fall within Kirkpatrick Levels 1 and 2, indicating a positive reaction from participants and an improvement in learning, though few reach Level 3, which assesses behavior change.

Future studies should aim to explore the application and impact of ERs in lower-income settings, veterinary education, and beyond the pre-clinical and clinical focus. This expansion could provide valuable insights into the versatility and adaptability of ERs as educational tools. There is a clear need for more robust study designs, including randomized controlled trials, to strengthen the evidence base for ERs in medical education. Additionally, longitudinal studies assessing Level 3 and Level 4 outcomes of the Kirkpatrick Model would provide a more comprehensive understanding of the impact of ERs on behavior change and clinical practice.

### Strengths and limitations

The use of a formulated protocol, and PRISMA guidelines for conducting and reporting systematic reviews is considered a strength of this article. Furthermore, the search string and terms have been reviewed and validated by a team of librarians at NTNU and were involved in the initial stages of the review. The topic is also a crucial and emerging field what has the potential to have impact on the implementation and design of ER in education.

The variety of evaluation formats (interviews, surveys, single-best answer questions etc.) and their accompanied reporting styles limit the ability for a meta-analysis due to inherent different in data types, metrics and reporting styles. Different evaluation formats often use different metrics for reporting results; inconsistency in the depth and format of the reporting styles means that the same concept (e.g., student satisfaction or learning outcomes) can be captured in different ways, making it difficult to draw direct comparisons across studies. There is also likelihood of publication bias, since all the studies reported improvements in knowledge, attitudes, and skills [84]. Articles with negative outcomes are less likely to be reported or accepted for publication [84]. Conference abstracts with no full texts were excluded, allowing for the possibility of additional ongoing experiments that have yet to publish empirical data.

## Conclusions

Our review found that ERs are a valuable educational tool in the context of medical training, with the potential to enhance knowledge retention, engage students in active learning, increase satisfaction with learning experiences, and develop essential professional skills. However, the field is still in its infancy, with significant gaps in research methodology, geographic representation, and educational outcomes. Addressing these challenges through future research will be crucial for optimizing the design, implementation, and evaluation of ERs in medical and healthcare education contexts.

## Electronic Supplementary Material

Below is the link to the electronic supplementary material.

Supplementary Material 1

## Data Availability

No datasets were generated or analysed during the current study.

## Abbreviations

ERs: Escape rooms
AMR: Antimicrobial resistance
PRISMA: Preferred Reporting Items for Systematic Reviews and Meta-Analysis
PICO: Population, Intervention, Comparator and Outcome
RQ: Research question
MERSQI: Medical Education Research Study Quality Instrument
HICs: High-income countries
IPP: Interprofessional practice
CoPs: Communities of Practice
