# Supplementary material for: Escaping the ordinary: a review of escape rooms in medical and veterinary education
Source: BMC Med Educ. 2024 Dec 20;24:1506. doi: 10.1186/s12909-024-06512-w (PMC11660942; doi:10.1186/s12909-024-06512-w)
Supplement: Supplementary file 1 — Supplementary Material 1 [file 12909_2024_6512_MOESM1_ESM.docx]

**Appendix 1. Search details.**

PubMed*

| Row | Query | Results |
| --- | --- | --- |
| 1 | "escape room"[tiab:~2] | 212 |
| 2 | "escape game" | 16 |
| 3 | “escape room*” | 223 |
| 4 | #1 OR #2 OR #3 | 243 |
| 5 | Education, Professional[MeSH Terms] | 336,133 |
| 6 | Teaching[Mesh Terms] | 94,080 |
| 7 | “teaching” | 2 022 061 |
| 8 | “training” | 2 702 627 |
| 9 | “pedagog*” | 21 413 |
| 10 | Academic Success[MeSH Terms] | 2 488 |
| 11 | Academic Performance[MeSH Terms] | 4, 337 |
| 12 | “learning outcome” | 117 031 |
| 13 | #5 OR #6 OR #7 OR #8 OR #9 OR #10 OR #11 OR #12 | 2 938 218 |
| 14 | **#4 AND #13** | **216** |

Embase*

| **Row** | **Query** | Results |
| --- | --- | --- |
| **#1** | **'escape room*'** | 285 |
| **#2** | **'escape game*'** | 17 |
| **#3** | **#1 OR #2** | 297 |
| **#4** | **‘education’ /exp** | 2 367 844 |
| **#5** | **‘teaching’/exp** | 116 658 |
| **#6** | **‘teaching’** | 443 811 |
| **#7** | **'training'** | 1 055 487 |
| **#8** | **‘pedagog*’** | 27 260 |
| **#9** | **‘Academic success’ /exp** | 4 173 |
| **#10** | **‘Academic achievement’ /exp** | 49 424 |
| **#11** | **‘learning outcome’** | 861 |
| **#12** | **#4 OR #5 OR #6 OR #7 OR #8 OR #9 OR #10 OR #11** | 3 377 588 |
| **#13** | **#3 AND #12** | **265** |

/ = Emtree term

Cochrane

| **Row** | **Query** | Results | |
| --- | --- | --- | --- |
| **#1** | **'escape room*'** | 85 | |
| **#2** | **'escape game*'** | 37 | |
| **#3** | **#1 OR #2** | 106 | |
| #4 | MeSH descriptor: [Education, Professional] explode all trees | 7203 |  |
| #5 | MeSH descriptor: [Teaching] explode all trees | 5 890 |  |
| #6 | “teaching” | 23 633 |  |
| #7 | “training” | 136 229 |  |
| #8 | “pedagog*” | 770 |  |
| #9 | Academic Success[MeSH Terms] | 118 |  |
| #10 | Academic Performance[MeSH Terms] | 226 |  |
| #11 | “learning outcome” | 13 428 |  |
| #12 | #4 OR #5 OR #6 OR #7 OR #8 OR #9 OR #10 OR #11 | 164 399 |  |
| #13 | | **#3 AND #12** | **53** |
| Scopus | |  |  |
| **Row** | **Query** | **Results** | |
| **#1** | **TITLE-ABS-KEY ( "escape room*" )** | 783 | |
| **#2** | **TITLE-ABS-KEY ( "escape game*" )** | 143 | |
| **#3** | **#1 OR #2** | 888 | |
| #4 | TITLE-ABS-KEY ( teaching ) | 812 497 |  |
| #5 | TITLE-ABS-KEY ( training ) | 1 704 877 |  |
| #6 | TITLE-ABS-KEY ( pedagog* ) | 194 285 |  |
| #7 | TITLE-ABS-KEY ( "learning outcome" ) | 41 996 |  |
| #8 | #4 OR #5 OR #6 OR #7 | 2 512 847 |  |
| #9 | | **#3 AND #8** | **405** |
